# An open-label randomised controlled trial evaluating the efficacy of a meningococcal serogroup B (4CMenB) vaccine on *Neisseria gonorrhoeae* infection in gay and bisexual men: the MenGO study protocol

**DOI:** 10.1186/s12889-023-15516-y

**Published:** 2023-03-30

**Authors:** Caroline Thng, Evgeny A. Semchenko, Ian Hughes, Maree O’Sullivan, Kate L. Seib

**Affiliations:** 1 Gold Coast Sexual Health, Southport Community Health Precinct, Southport, Qld 4215 Australia; 2 Institute for Glycomics, Griffith University, Southport, Qld 4215 Australia; 3 Gold Coast University Hospital, Southport, Qld 4215 Australia

**Keywords:** Gonorrhoea, *Neisseria gonorrhoeae*, Sexually transmitted infection (STI), Gay and bisexual men (GBM), Men who have sex with men (MSM), 4CMenB, Bexsero, Vaccine

## Abstract

**Background:**

Gonorrhoea is an ongoing public health concern due to its rising incidence and the emergence of antibiotic resistance. There are an estimated 82 million new *Neisseria gonorrhoeae* infections each year, with several populations at higher risk for gonococcal infection, including gay and bisexual men (GBM). If left untreated, infection can lead to serious morbidity including infertility, sepsis and increased risk of HIV acquisition. Development of a gonorrhoea vaccine has been challenging, however there is observational evidence that serogroup B meningococcal vaccines, used to protect against the closely related bacteria *Neisseria meningitidis*, could provide cross-protection against *N. gonorrhoeae.*

**Methods:**

The MenGO (Meningococcal vaccine efficacy against Gonorrhoea) study is a phase III open-label randomised control trial in GBM to evaluate the efficacy of the four-component meningococcal serogroup B vaccine, 4CMenB, against gonorrhoea. A total of 130 GBM will be recruited at the Gold Coast Sexual Health Clinic, Australia, and randomised to either receive 2 doses of 4CMenB or no intervention. Participants will be followed up for 24 months with testing for *N. gonorrhoeae* and other sexually transmissible infections every three months. Demographics, sexual behaviour risk, antibiotic use, and blood samples for analysis of *N. gonorrhoeae-*specific immune responses, will be collected during the study. The primary outcome is the number of *N*. *gonorrhoeae* infections in participants over 2 years measured by nucleic acid amplification test (NAAT). Secondary outcomes are vaccine-induced *N. gonorrhoeae-*specific immune responses, and adverse events in trial participants.

**Discussion:**

This trial will determine if the 4CMenB vaccine is able to reduce *N. gonorrhoeae* infection. If shown to be effective, 4CMenB could be used in gonococcal prevention. Analysis of 4CMenB-induced immune responses will increase understanding of the type of immune response needed to prevent *N. gonorrhoeae*, which may enable identification of a potential correlate of protection to aid future gonorrhoea vaccine development.

**Trial registration:**

The trial has been registered on the Australian and New Zealand Clinical Trials Registry (ACTRN12619001478101) on 25 October 2019.

**Supplementary Information:**

The online version contains supplementary material available at 10.1186/s12889-023-15516-y.

## Background

*Neisseria gonorrhoeae* is a significant healthcare challenge worldwide, causing an estimated 82 million cases/year of the sexually transmitted infection (STI) gonorrhoea [1]. Gonorrhoea cases have risen in many regions in recent years (e.g., 45% increase in the USA in the 5 years between 2016 and 2020 [2] and 144% increase in Australia in the 10 years between 2011 and 2020 [3]). Gonorrhoea infection is more prevalent in particular risk groups, including gay and bisexual men (GBM) [4]. GBM who are taking HIV pre-exposure prophylaxis (PrEP) have an incidence rate of 37.8 cases per 100-person years [5]. Gonorrhoea is increasingly difficult to treat due to ongoing emergence of antimicrobial resistance [6]. *N. gonorrhoeae* has developed resistance to all classes of antibiotics used to treat it since the 1940s, with new treatment regimens needed every 5–10 years [7]. In particular, treatment for pharyngeal gonorrhoea remains a challenge, with lower treatment efficacy for current and new antibiotic regimens [8], contributing to the emergence of antibiotic resistance and onward transmission. Since 2013, the WHO and CDC have highlighted gonorrhoea as an “urgent” public health threat [9–11] and in 2018, ‘super gonorrhoea’, resistant to all routine antibiotics was reported in the UK [12] and Australia [13].

*N. gonorrhoeae* infection can result in a range of clinical outcomes, which vary by site of infection and by sex [14]. Pharyngeal and anal infections are typically (~ 90%) asymptomatic, while genital tract infection is more frequently symptomatic. In men, genital infection is typically characterised by symptomatic urethritis, but may be asymptomatic in up to 10% of patients [15]. Complications of untreated infection in men include urogenital tract abscesses, orchitis, and prostatitis [16]. In women, 50–80% of lower genital tract gonorrhoea infections remain asymptomatic, and hence are frequently untreated. Ascending infection to the Fallopian tubes occurs in up to 45% of infected women, and can result in serious complications including pelvic inflammatory disease, ectopic pregnancy, spontaneous abortion, pre-term or low weight birth, stillbirth, neonatal blindness and infertility [16]. Gonorrhoea increases the risk of HIV transmission, and is a major contributor to the ongoing HIV epidemic [14]. Hence, the development of a vaccine to prevent gonorrhoea is urgently needed [9] .

Despite more than a century of research, there is currently no gonorrhoea vaccine. This is largely due to the fact that gonococcal infection does not protect against subsequent infection, therefore there are no correlates of protection from natural immunity to guide vaccine development [14]. Moreover, humans are the only natural host for *N. gonorrhoeae* and there is no animal model that accurately mimics human infection and transmission. Thus, there are limited tools to enable discovery and evaluation of potentially protective vaccine candidates [14]. However, it has been reported that meningococcal serogroup B vaccines, used to protect against the closely related bacteria *Neisseria meningitidis*, may be associated with reduced incidence of *N. gonorrhoeae* infection [17–20].

The meningococcal B outer membrane vesicle (OMV) vaccine MeNZB™ was used to vaccinate > 1 million people in New Zealand between 2004–2008 in response to a meningococcal B epidemic [21]. A retrospective case-control study of this cohort showed that MeNZB™-vaccinated individuals were significantly less likely to contract gonorrhoea compared to unvaccinated controls, with a predicted vaccine efficacy of 31% [17]. Although modest, this was the first time a vaccine has been associated with any protection against gonorrhoea [18]. A broader spectrum four-component meningococcal B vaccine, 4CMenB (marketed as Bexsero®) is now available and contains the MeNZB™ OMVs plus three recombinant protein antigens (NadA, and the fHbp-GNA2091 and NHBA-GNA1030 fusions) [22]. Recent observational cohort studies using data linkage have demonstrated 4CMenB vaccine efficacy of 32–40% in preventing gonorrhoea infection in young adults [19, 20]. Mathematical modelling of hypothetical gonococcal vaccines has indicated that even a vaccine with a modest effectiveness of ~ 30% could reduce the population prevalence of gonorrhoea by ~ 50% within 20 years in a general heterosexual population [23] and by > 40% within 2 years in a GBM population [24].

The level of antigenic identity between *N. meningitidis* and *N. gonorrhoeae* has been suggested to be responsible for the possible cross protection between the meningococcal serogroup B vaccines and *N. gonorrhoeae.* Bioinformatic analysis has identified that a homologue of 20 of the 22 major meningococcal OMV proteins on the 4CMenB vaccine are present in *N. gonorrhoeae* (16 proteins have > 90% identity, 2 proteins have > 80% identity to the meningococcal vaccine antigen), and the 4CMenB NHBA antigen shares > 67% identity to NHBA from *N. gonorrhoeae* strains [25]. Furthermore, human anti-4CMenB antibodies are cross-reactive with *N. gonorrhoeae* [25]. The 4CMenB vaccine has been licensed in more than 40 countries worldwide, and the safety and side effects of the vaccine have been well documented [26]. 4CMenB is licensed in Australia for active immunisation against invasive disease caused by *N. meningitidis* group B strains in people > 2 years of age (two doses with an interval of ≥ 1 month between doses) [27].

## Methods

### Aims and hypotheses

The MenGo trial aims to determine if vaccination with the meningococcal vaccine 4CMenB can reduce the incidence of *N. gonorrhoeae* infection in GBM over 2 years, and to investigate vaccine induced immune responses during the study period. We hypothesise that 4CMenB could reduce the risk of acquiring gonorrhoea and that investigation of 4CMenB-induced antibodies that are cross-reactive with *N. gonorrhoeae* could reveal the mechanism of 4CMenB-induced protection against *N. gonorrhoeae* infection.

### Study design and setting

**Fig. 1:**
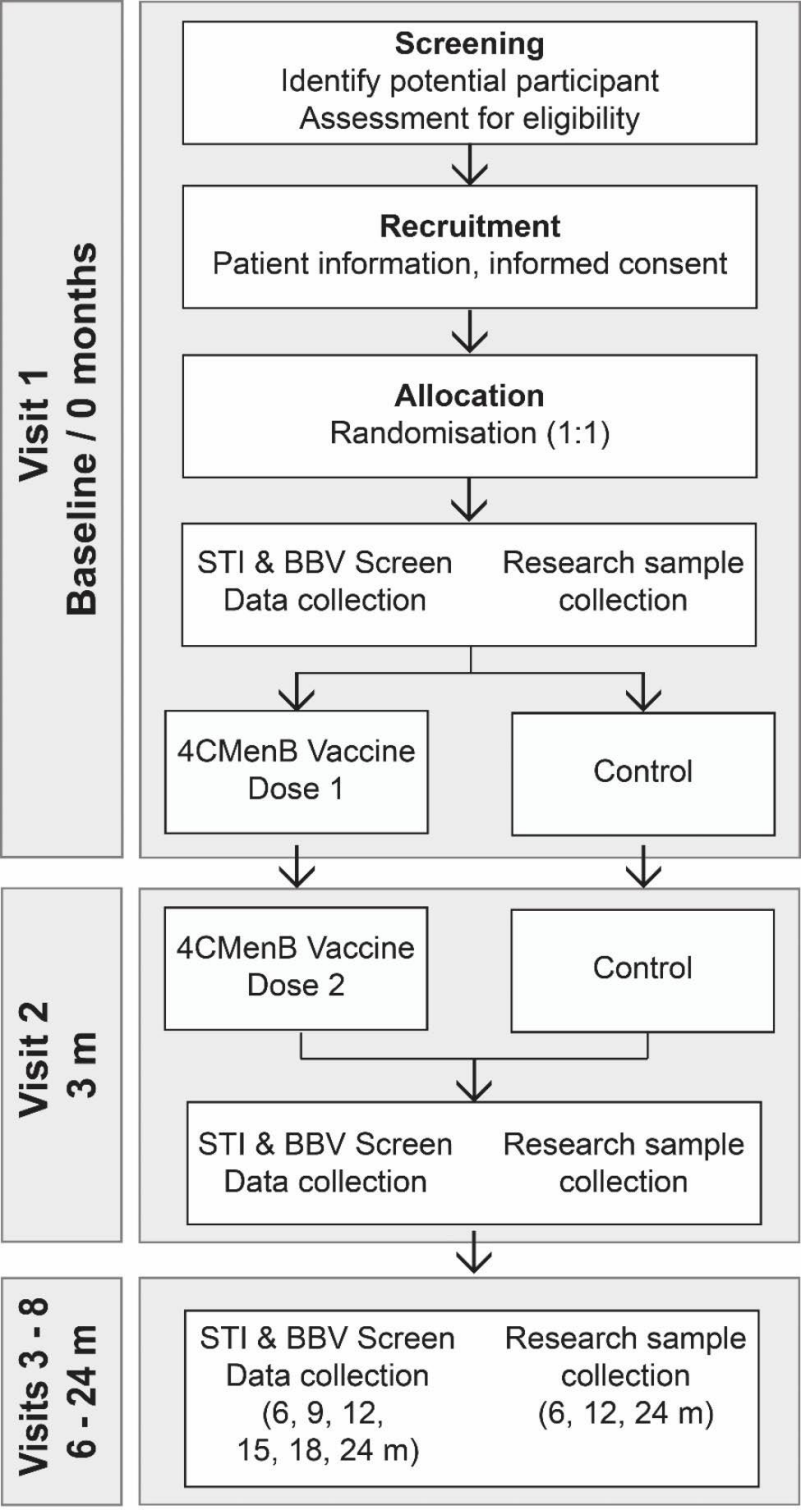
Overview of MenGO Trial design

MenGO is a Phase III open-label unblinded randomised control trial of 4CMenB vaccination of GBM to protect against *N. gonorrhoeae* infection. The study design is shown in Fig. 1. Participants will be randomly assigned by 1:1 allocation to either: (1) the ‘4CMenB vaccine arm’ who will be vaccinated at baseline and 3 months with 4CMenB; or (2) the unvaccinated ‘no treatment arm’. An STI screen as per the Australian STI guidelines [28] will occur at baseline and at 3 monthly study visits over 2 years. Blood will be collected for antibody analysis at 0, 3, 6, 12, and 24-month visits, and information regarding demographics, concurrent medication, previous gonorrhoea infections, antibiotic use and sexual behaviour risks will be collected at each visit. The trial will be conducted in accordance with the guidelines for Good Clinical Practice (GCP) and will satisfy Consolidated Standards of Reporting Trials (CONSORT) reporting conventions [29].

### Eligibility criteria for participants

The study is limited to GBM attending a public sexual health clinic, who are at a high and ongoing risk of acquiring *N. gonorrhoeae*.

#### Inclusion criteria

GBM who are:

i. Between 18 and 50 years of age.
ii. Able to understand spoken and written English.
iii. Men who have sex with other men.
iv. Able to participate in study procedures including attending for all study visits.
v. At risk of acquisition of gonorrhoea at the time of enrolment into the study.
vi. Agree to be contacted via phone/ email by the study team.

AND are:

- Currently taking HIV pre-exposure prophylaxis (PrEP) at the time of enrolment OR
- Have been diagnosed with gonorrhoea in the last 3 months.

#### Exclusion criteria

Participants will be excluded from participation if they have:

i. Documented allergy to latex and/ or kanamycin.
ii. Confirmed previous history of vaccination for meningococcal B with 4CMenB.
iii. Contraindications to receiving the meningococcal B vaccine which include:

- Anaphylaxis following a previous dose of any meningococcal vaccine.
- Anaphylaxis following any vaccine component.

iv. Participants who are currently recommended and funded to receive 4CMenB [30] including participants who have:

- Sickle cell disease or other haemoglobinopathies.
- Congenital or acquired asplenia (e.g., plenectomy or hyposplenia).
- Defects in, or deficiency of, complement components including factor H, factor D or properdin deficiency.
- Current or planned future treatment with eculizumab (a monoclonal antibody directed against complement component C5).

Previous meningococcal disease and previous vaccination with the strain specific meningococcal B vaccine MeNZB™ is not a contraindication for receiving 4CMenB.

### Intervention

Participants will be randomly assigned to either the ‘4CMenB vaccine arm’ or the ‘no treatment arm’ of the study. Participants allocated to the vaccine group will receive a 2-dose schedule of 4CMenB (first dose at baseline visit, second dose at 3-month visit). For each dose, 0.5mL of 4CMenB will be administered by deep intramuscular injection, preferably in the deltoid muscle region of the upper arm. Participants allocated to the no treatment control group will not receive any treatment.

### Outcomes

Table 1 lists primary and secondary outcomes alongside key study objectives and endpoints.

#### Primary outcome

(i) Number of *N*. *gonorrhoeae* infections in participants at any anatomical site over 2 years, diagnosed by nucleic acid amplification test (NAAT).

#### Secondary outcomes

(ii) Level and function of *N. gonorrhoeae-*specific antibodies.
(iii) Number of serious adverse events (SAEs) reported where the cause has been determined to be related to the study treatment.
(iv) Number of participants consented to the trial compared to number of participants where pre-screening was performed.

**Table 1:** Study objectives, outcomes and endpoints

Objectives	Outcome / Endpoint
**Primary**	
Determine if vaccination with 4CMenB reduces the incidence of gonorrhoea	Number of *N*. *gonorrhoeae* infections at any anatomical site over 2 years, diagnosed by nucleic acid amplification test (NAAT).
**Secondary**	
Characterise humoral immune response in 4CMenB-vaccinated serum	Level and function of *N. gonorrhoeae-*specific antibodies.
Determine the safety and tolerability of 4CMenB in GBM aged 18–50 years	Number of SAEs reported where the cause has been determined to be related to the study treatment
Determine the uptake and feasibility of 4CMenB as a preventative strategy for gonorrhoea in GBM attending sexual health services	Number of participants consented to the trial compared to number of participants where pre-screening was performed

### Sample size

A target of 130 participants will be recruited into the study. To detect a reduction in gonococcal prevalence from 30% (based on a gonococcal prevalence of 29.4% in patients taking PrEP at Gold Coast Sexual Health Service) to 10% (assuming a vaccine efficacy of 30%, based on the predictive MeNZB™ [17]) with 80% power at a significance level of 0.05 using the proportions test, 112 participants will need to be recruited into equal groups of 56 (G*Power 3.1). To account for an attrition or loss to follow up at a rate anticipated to be less than 10% the total recruiting target is 130. The Gold Coast Sexual Health Service see more than 10,000 patients per annum of which approximately 1,700 are GBM, which should enable adequate recruitment to reach the target sample size.

### Recruitment and consent

Potentially eligible participants will be identified by clinicians and will be referred to a study investigator who is delegated to provide further information and check eligibility. Eligible participants will be given a Participant Information and Consent Form (PICF) and asked to provide written informed consent prior to commencement in the study. Participants will also consent for the study team to acquire results from other health services for the purpose of the research, to maximise data collection should participants attend for gonorrhoea testing elsewhere during the period of the study. Participants will be able to change or withdraw their consent to participate at any time during the course of the study.

### Interventions

The MenGo trial involves nine study visits over 24 months for each participant, including a baseline visit (month 0) and 3-monthly follow up visits. Table 2 outlines the trial schema. Participants in the ‘4CMenB vaccine arm’ will receive vaccination at baseline (dose 1) and at month 3 (dose 2). Participants who receive the vaccine will have their blood pressure and temperature taken prior to administration of vaccine. All other physical examinations at all time points will be guided by patient symptoms. At baseline and at each 3-monthly follow up visit, participants in both the ‘4CMenB vaccine arm’ and the ‘no treatment arm’ groups will undergo a sexual health screen as per the Australian STI guidelines [28]. Samples taken will include first-void urine for urogenital gonorrhoea and chlamydia by NAAT, oropharyngeal and anorectal swabs for gonorrhoea and chlamydia by NAAT, and blood for HIV, hepatitis and syphilis screening. Urine and swabs for microscopy examination, *Mycoplasma genitalium* testing and other microbiological culture and sensitivity testing may also be collected. Participants with a positive test will be recalled to the clinic for management and treatment as per standard of care. Participants will continue to receive other standard of care testing and treatment as required. A non-standard-of-care blood sample will be collected from both groups at 3, 6, 12 and 24 months post vaccine for analysis of *N. gonorrhoeae-*specific antibodies.

In order to maximise retention in the study, all participants will receive an automated reminder of their next study visit via the clinic electronic reminder system. Participants will routinely be sent an SMS or email reminder of appointments. Participants who failed to attend will be sent two reminders via SMS or email and a final phone call. Unless a participant is discontinued from the study, study visits, procedures and data can continue to be collected at any time from enrolment up to 24 months (+ 14 days). Reasons for discontinuation include participant withdrawal, adverse event, investigator discretion or termination of the study by the Sponsor. Participants who are lost to follow up during the study period will not be replaced.

**Table 2:** Study Schema

Month	0 *	3 *	6 *	9*	12 *	15 *	18 *	21 *	24 *	Un-scheduled
Eligibility	√									
Consent	√									
Randomisation	√									
Vaccination	√	√								
Physical examination (BP, temp)	√	√								
Risk Assessment *	√	√	√	√	√	√	√	√	√	√
Sexual health & blood-borne virus test and results *	√	√	√	√	√	√	√	√	√	√
Research blood sample	√	√	√		√				√	
Safety assessment for adverse events	√	√	√	√	√	√	√	√	√	√

*indicates standard of care visits and procedures for participants currently taking PrEP. Study tests and procedures which have been taken within 14 days of the study visit that have already been taken as standard of care for the patient, can be used for study and do not need to be repeated.

### Randomisation and allocation

Participants will be randomized 1:1 to 2 groups of 65 – the ‘4CMenB vaccine arm’ or the ‘No vaccine arm’. A biostatistician independent of the clinical researchers will use the ralloc package of Stata 17 (College Station, Tx., USA) to perform block randomization with blocks of size 2–6. Allocations will be placed in sequentially numbered sealed envelopes and kept in a locked cupboard with restricted access to the area (security pass protected). Randomised allocation will only be performed once written informed consent has been obtained. Investigators will allocate study participants from the sequential sealed envelopes, ensuring that there is blinding of the randomisation process.

### Sample processing and testing

All samples for tests which participants receive as standard of care will be transported to Pathology Queensland (Gold Coast University Hospital) for testing according to existing local procedure and policy. For *N. gonorrhoeae* detection by NAAT, all specimens will undergo the combined *Chlamydia trachomatis/ Neisseria gonorrhoeae* test on the Roche Cobas® 4800 machine which is currently used as standard of care by Pathology Queensland. Oropharyngeal and anorectal samples which test positive are confirmed by a secondary Pathology Queensland in-house PCR targeting the gonococcal *opa* genes and *porA* pseudogene [31]. Results from NAAT are reported as *N. gonorrhoeae* “Detected” or “Not detected”. *N. gonorrhoeae* infection is diagnosed only if both the Roche Cobas® 4800 and in-house confirmatory tests are reported as “Detected”. Culture will also be performed for any site which is positive by NAAT for antimicrobial susceptibility testing.

The non-standard of care blood sample for *N. gonorrhoeae-*specific antibody assays will be allowed to clot, then centrifuged at 2,000 x g for 10 min at the Gold Coast Sexual Health Clinic. Serum will be aliquoted into sterile tubes and stored at − 20˚C prior to transport to the Institute for Glycomics, Griffith University for analysis as per standard methods [25, 32–35]. Antibodies in blood-derived serum will be characterised using enzyme-linked immunosorbent assay (ELISA) to quantify the level of specific antibody isotypes that cross-react with *N. gonorrhoeae* whole cell bacteria, and specific target proteins) [25, 32–35]. The ELISA titre is defined as the reciprocal of highest serum dilution with a positive signal (i.e., a signal greater than the negative sample mean plus three standard deviations). The functional activity of antibodies against *N. gonorrhoeae* will be tested in serum bactericidal activity (SBA) and neutrophil-dependent opsonophagocytic activity (OPA) assays [32–35]. Antibody neutralisation of *N. gonorrhoeae* may also be tested in functional assays that measure antibody-dependent blocking of bacterial adherence to epithelial cells or of antigen binding to their ligand [33, 34]. The SBA, OPA or neutralisation titre is defined as the reciprocal of the final serum dilution giving ≥ 50% killing or neutralisation.

## Data collection and management

Data collected at each visit will include patient demographics, concurrent medication, previous *N. gonorrhoeae* infections, antibiotic use, sexual behaviour risks and previous STI and blood borne virus (BBV) testing within the last 3 months. If the participant has had testing for *N. gonorrhoeae* elsewhere, all efforts will be made to obtain a copy of those results and associated clinical information either from the patient or directly from the treating physician or clinic. If the participant reports any antibiotic use, the name of the antibiotic and reason for use will also be asked.

### Adverse events

Adverse events (AE) and serious adverse events (SAEs), according to the Common Terminology Criteria for Adverse Events (CTCAE), will be collected from the participant self-reported diary and adverse event enquiry by study investigators at each visit. All SAEs will be reported as per Therapeutic Goods Administration and the Gold Coast Hospital Ethics Committee guidelines.

### Data management

Clinical data and all data in case report forms will be entered into the Sexual Health Information Programme (SHIP) which is the study site’s clinical electronic medical record software, RedCap® and Microsoft Excel. All databases will be stored on a Queensland Health, password protected secure network. Upon consent to participate in the study, the participant will be allocated a study number which will be used to identify the participant throughout the study and retain participant confidentiality. A Master log will be kept for re-identification in the case where participants are required to be re-called for study, safety or clinical reasons. All data will be deidentified for monitoring and audit purposes (by a Study Monitor from the Office of Research, Governance and Development at the Gold Coast University Hospital) and for presentation or publication of results. The Sponsor will also be advised by a Data and Safety Monitoring Committee made up of independent members not involved in the study.

### Statistical methods

Data analysis will be performed by statisticians at the Gold Coast Hospital and Health Service (GCHHS) who are not involved in other study procedures, to reduce chance of bias in the statistical analysis. The number and percentage of participants with NAAT confirmed *N. gonorrhoeae* from any anatomical site will be presented along with 95% confidence interval (CI) at the completion of the study by treatment group. Total observation time will also be recorded. Associations between 4CMenB vaccination status and the incidence of *N. gonorrhoeae* infection will be investigated using Fisher’s exact test and univariate Poisson regression. The incident rate ratio (IRR) with 95% CIs of incidence of *N. gonorrhoeae* infection in the vaccinated group compared to the unvaccinated group will be calculated. Demographic and clinical characteristics of the 4CMenB vaccinated group and the “no vaccine” group will be compared. If any variables are seen to be different between groups (not expected due to randomization) these will be included in a multivariable Poisson regression model along with 4CMenB vaccine predicting *N. gonorrhoeae* infection. The difference in incidence rates of *N. gonorrhoeae* infections between the study treatment and no treatment at the end of the 2-year study period will also be calculated along with 95% CI. The study treatment will be considered effective if the lower limit of the 2-sided 95% CI for the difference between the incidence rates of gonorrhoea in the study treatment group is 30% lower than the no treatment group. Further analyses may be performed based on the observed differences between the treatments in some subgroups. If it is the case that individual participants record more than one incidence of *N. gonorrhoeae* infection in the period of the study, the same analyses will be performed but using a generalized estimating equations (GEE) approach to Poisson regression to account for within participant correlation.

Log(base2) transformed antibody, SBA, OPA and neutralisation titres for each of the 0, 3, 6, 12, and 24-month time points will be, initially, compared between vaccinated and non-vaccinated groups by linear regression with log_2_-titre at time 0 as a covariate. Other clinical and demographic covariates will also be tested, along with vaccination status. Variables will be considered as covariates and retained in the model if P < 0.05. Finally, a mixed effects analysis of the trajectory of the log_2_-titres over the 24-month duration of the study will be performed with participant number as a random effect and vaccination status as the fixed effect of interest. Again, other clinical and demographic variables will be tested as covariates.

The number and percentage of treatment-emergent AEs, study treatment-related AEs, SAEs and AEs leading to study withdrawal will be provided.

## Discussion

A vaccine to protect against *N. gonorrhoeae* infection is urgently needed due to the rising gonorrhoea case numbers and the continued emergence of antibiotic resistance. Despite more than a century of research, only four gonococcal vaccine candidates have been tested in human clinical trials and none of these elicited protection against gonorrhoea [14]. Failures of these early vaccine candidates created hesitancy in the field and delayed gonococcal vaccine development. However, the field has been reinvigorated by recent epidemiological [17, 19, 20, 36–39] and in vitro data [40] suggesting that meningococcal B vaccines may provide cross protection against gonorrhoea.

MenGO is one of the first randomised control trials worldwide that aims to determine the efficacy of the licenced meningococcal B vaccine 4CMenB against gonorrhoea. The trial includes GBM on PrEP, or GBM with a recent *N. gonorrhoeae* infection. This study is limited to GBM due to the high gonorrhoea incidence in this cohort. In Australia, the majority of *N. gonorrhoeae* infections are in GBM [3], and GBM on PrEP have a gonorrhoea incidence of 37.8 cases per 100-person years [5]. Current guidelines for participants taking PrEP recommend 3-monthly review by a health practitioner for testing for STIs [41]. Therefore, participants are unlikely to bear an excess burden to participate in this study. Recent, prior infection with *N. gonorrhoeae* is a risk factor associated with STI diagnosis [42] and is therefore added as an inclusion criterion.

If this study shows that the 4CMenB vaccine is effective in reducing the incidence of gonorrhoea in GBM, this will be the first direct evidence that vaccination can prevent *N. gonorrhoeae*. The opportunity to use the licenced 4CMenB vaccine would be a relatively rapid and cost-effective approach to reduce gonorrhoea prevalence. Given the high gonorrhoea incidence in GBM, combined increased risk of HIV acquisition associated with concurrent *N. gonorrhoeae* infection [43, 44] and the current threats to management of gonorrhoea due to the emergence of multi-drug resistance [45] new prevention strategies are urgently needed in high risk populations such as GBM. Use of 4CMenB in GBM would also impact the wider population due to bridging between GBM and heterosexuals [46, 47]. Furthermore, outcomes of this study would aid policy and practice change to facilitate the investigation and use 4CMenB as a general population gonorrhoea prevention strategy.

The type of the immune response needed to prevent infection with *N. gonorrhoeae* is unknown, and investigation of the immune response to 4CMenB in this study will enable characterisation of antibodies that are cross-reactive with *N. gonorrhoeae.* This may enable identification of the mechanism of vaccine-mediated protection and of a correlate of immune protection. This aspect of the study is unique and of critical importance to enable ongoing evaluation of 4CMenB cross-protection and to facilitate the development of a gonorrhoea vaccine with higher efficacy and/or a different product profile if required.

If 4CMenB is effective against *N. gonorrhoeae*, this could lead to a widespread change in prevention and control strategies, which may lead to a substantial decrease in gonorrhoea. This would improve sexual and reproductive health and reduce costs associated with testing, treatment and management of *N. gonorrhoeae*-associated antimicrobial resistance and sequelae.

## Electronic supplementary material

Below is the link to the electronic supplementary material.

Supplementary Material 1

## Data Availability

Data sharing does not apply to this article as data collection has not yet been completed or analysed.

## List of abbreviations

4CMenB: four-component meningococcal B vaccine
ELISA: enzyme-linked immunosorbent assay
GBM: gay and bisexual men
HIV: Human immunodeficiency virus
HREC: Human Research Ethics Committee
MSM: Men who have sex with men
NAAT: Nucleic acid amplification test
OPA: Opsonophagocytic activity
PrEP: Pre-exposure prophylaxis
RCT: Randomised controlled trials
SBA: serum bactericidal activity
STI: Sexually transmissible infection.
